# A Streamlined Approach to Anticounterfeiting Technologies: Patterned AAO Membranes Based on Photonic Crystal Effects with Tunable Color Shifts and pH Responsiveness

**DOI:** 10.1002/smll.202409919

**Published:** 2025-01-14

**Authors:** Yu‐Chun Lin, Lin‐Ruei Lee, Tsung‐Hung Tsai, Ji Lin, Yen‐Shen Hsu, Manibalan Kesavan, Yu‐Liang Lin, Yi‐Fan Chen, Jiun‐Tai Chen

**Affiliations:** ^1^ Department of Applied Chemistry National Yang Ming Chiao Tung University Hsinchu 300093 Taiwan; ^2^ Center for Emergent Functional Matter Science National Yang Ming Chiao Tung University Hsinchu 300093 Taiwan

**Keywords:** anodic aluminum oxide, anticounterfeiting, flexible, photonic crystal, pH‐responsive

## Abstract

Anticounterfeiting technologies have become increasingly crucial due to the growing issue of counterfeit goods, particularly in high‐value industries. Traditional methods such as barcodes and holograms are prone to replication, prompting the need for advanced, cost‐effective, and efficient solutions. In this work, a practical application of anodic aluminum oxide (AAO) membranes are presented for anticounterfeiting, which addresses the challenges of high production costs and complex fabrication processes. Unlike previous approaches requiring metal coatings for color generation, this method uses commercial aluminum foils to produce colorful AAO membranes without metal layers. Elemental mapping suggests that impurities on the aluminum surface contribute to enhanced reflectivity, aiding photonic crystal formation. A two‐step anodization process that creates patterned AAO membranes is further introduced, with the pattern clarity controlled by anodization time. Additionally, a pH‐responsive film composed of 2‐anilino‐6‐dibutylaminofluoran (ODB‐2) and thermoplastic polyurethane (TPU) is integrated, enabling dynamic color changes under varying pH conditions, further enhancing the anticounterfeiting functionality. This streamlined approach provides a scalable and cost‐effective solution for developing versatile AAO membranes for industrial anticounterfeiting applications.

## Graphical abstract

A practical application of anodic aluminum oxide (AAO) membranes for anticounterfeiting is presented. Unlike previous approaches requiring metal coatings for color generation, this method uses commercial aluminum foils to produce colorful AAO membranes without metal layers. A two‐step anodization process that creates patterned AAO membranes is further introduced, with the pattern clarity controlled by anodization time.

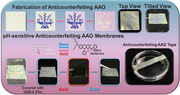

## 1 Introduction

The issue of counterfeit goods has grown increasingly severe, significantly disrupting the global market, particularly in high‐value sectors such as cosmetics, pharmaceuticals, electronics, and luxury goods.^[^ ^1^, ^2^, ^3^ ^]^ The development of anticounterfeiting technologies has become a crucial means of protecting product authenticity and preserving brand integrity. For the technologies to be effective, they must possess high accuracy in identification and be resistant to replication. Ideal anticounterfeiting materials should offer durability, low cost, and the ability to be authenticated multiple times, ensuring excellent performance in practical applications.^[^ ^4^, ^5^, ^6^ ^]^ However, traditional anticounterfeiting methods, such as barcodes, invisible inks, and holograms, are often prone to forgery and duplication, providing limited protection.^[^ ^7^, ^8^ ^]^ As a result, high‐security, multifunctional, and mechanism‐complex anticounterfeiting materials or technologies have become increasingly important.^[^ ^9^, ^10^, ^11^, ^12^, ^13^ ^]^ Among these, nanostructure‐based anticounterfeiting technologies have gained increasing attention.^[^ ^14^, ^15^, ^16^, ^17^ ^]^ Attributed to the complexity and unique properties of nanostructures, resultant patterns are challenging to replicate, offering higher levels of security and making these technologies suitable for advanced anti‐counterfeiting applications.

Among nanostructured materials, anodic aluminum oxide (AAO) membranes have been widely used and recognized.^[^ ^18^, ^19^, ^20^, ^21^ ^]^ The self‐ordered nanoporous structures of AAO membranes find extensive applications in various fields such as nanostructure synthesis, catalysis, and biosensing.^[^ ^22^, ^23^, ^24^ ^]^ Additionally, AAO membranes serve as effective templates because of their ability to control nanopore distribution, pore size, and spacing by adjusting fabrication parameters such as electrolyte concentration, anodizing voltage, and anodizing time. By precisely tuning these parameters, the anodizing process enables the fabrication of uniformly thick porous aluminum oxide membranes, with film thicknesses accurately controlled at the nanoscale. This level of control ensures consistent structural properties across the membrane, making them ideal for the production of various nanoscale structures.^[^ ^25^ ^]^ Moreover, in industrial applications, the demand for cost‐effective and efficient AAO fabrication techniques has become a critical focus of research.^[^ ^26^, ^27^, ^28^ ^]^

In the field of anticounterfeiting technology, AAO‐based technologies have been developed to create nanostructured security labels.^[^ ^29^, ^30^ ^]^ For example, Peng et al. utilized AAO membranes as templates to fabricate nanoimprint molds, which can be employed to produce polymer‐based anticounterfeiting labels through light interference in nanostructures;^[^ ^31^ ^]^ however, the use of nanoimprint technology and the introduction of polymers require specialized equipment and complex processes, increasing the overall cost of producing anticounterfeiting labels. In another example, Lee et al. employed AAO membranes grafted with a light‐responsive material, spiropyran, to create localized optical changes in the AAO membranes for anticounterfeiting applications;^[^ ^32^ ^]^ however, the need for grafted compounds makes the process complex and challenging for large‐scale production, limiting its range of applications. Both studies and other related works require complicated processes to apply AAO membranes in anticounterfeiting, which is not suitable for mass production.^[^ ^33^, ^34^ ^]^ Therefore, improving the fabrication process to make AAO membranes more accessible for anticounterfeiting applications remains a critical challenge.

To address the aforementioned challenges, we introduce an innovative and versatile approach to applying AAO membranes in the field of anticounterfeiting, which is based on the concept of photonic crystals on AAO membranes. The well‐ordered and uniformly deep nanopores of AAO membranes can behave as photonic crystals when exposed to light.^[^ ^35^, ^36^, ^37^ ^]^ Previous studies have shown that structural parameters such as pore size, thickness, and interpore distance of AAO membranes can affect light reflection properties, which in turn influence the color shifts in photonic crystals.^[^ ^38^, ^39^, ^40^ ^]^ Metal coatings, however, are usually required on AAO membranes to produce colors through light interference because of the transparency of aluminum oxide.^[^ ^29^, ^36^, ^41^, ^42^, ^43^ ^]^ In this work, we use commercial aluminum foils to fabricate AAO membranes that can produce colors from photonic crystals without the need for metal coatings. Elemental mapping analysis suggests that uniformly distributed impurities on the surface of the commercial aluminum foils might contribute to the reflectivity of the AAO membranes, enhancing the resultant colors. By applying a pulling machine, we also fabricate AAO membranes with gradient pore depths, which correlate with the anodization times, resulting in colorful surfaces. The simplified and efficient process reduces the production costs and only requires an anodization time of ≈10 min, enabling large‐scale fabrication of colorful anticounterfeiting AAO membranes.

For the anticounterfeiting applications, patterns are generated on the AAO membranes via a two‐step anodization method, where a hydrophobic mask is applied between the two anodization steps to create AAO membranes with two distinct pore depths. The clarity of the patterned AAO membranes can be controlled by adjusting the anodization times of the second step or varying the viewing angles. To enhance security and technical complexity, the integration of responsive materials with anticounterfeiting technologies has become a popular topic.^[^ ^44^, ^45^, ^46^ ^]^ Many responsive materials show great potential for anticounterfeiting applications.^[^ ^47^, ^48^, ^49^ ^]^ In this work, we apply a pH‐responsive 2‐anilino‐6‐dibutylaminofluoran (ODB‐2)/thermoplastic polyurethane (TPU) film onto the surface of the patterned AAO membranes,^[^ ^50^, ^51^, ^52^ ^]^ producing responsive anticounterfeiting AAO membranes. Under alkaline conditions, the ODB‐2/TPU film becomes transparent, revealing the designed patterns; while under acidic conditions, the film turns black, concealing the designed patterns. Furthermore, patterned AAO membranes are fabricated from commercial aluminum tapes, which can adhere to uneven surfaces, thereby enhancing the applicability of patterned AAO membranes in anticounterfeiting. We believe this research expands the versatility and flexibility of AAO membranes and offers new applications in the anticounterfeiting field.

## 2 Results and Discussion

**Figure 1:**
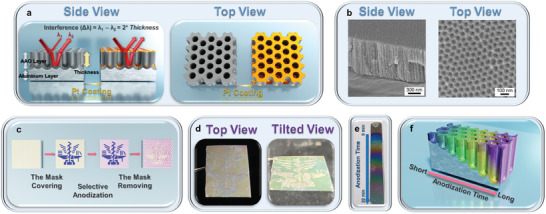
a) Schematic illustration of the side‐ and top‐views of AAO membranes. b) Side‐ and top‐view SEM images of AAO membranes. c) Schematic illustration of the fabrication process for patterned AAO membranes. d) Photos of patterned AAO membranes observed from the top and tilted views. e) Photo of an AAO membrane with gradient pore depths. f) Graphic illustration of an AAO membrane showing gradient pore depths corresponding to different anodization times.

**Figure** **1** demonstrates the principles and applications of the AAO membranes with thin‐film interference. Due to the porous structure of the AAO membranes, light creates two reflection paths on the surface of the AAO membranes, causing interference and resulting in visible wavelengths, as shown in Figure 1a. From the side‐view illustration, the AAO layer appears nearly transparent in the visible light range, while the aluminum layer is opaque. The two light paths consist of reflections at the AAO‐air interface (λ_1_) and the aluminum‐AAO interface (λ_2_). The wavelength of the thin‐film interference can be calculated using the Bragg reflector principle: Σ2n*_i_* d*_i_* cosθ*_i_* = *m*λ, where n*_i_*, d*_i_*, and θ*_i_* represent the effective refractive index, thickness, and reflection angle of layer *i*, respectively, while *m* and λ denote the interference order number and the wavelength of light, respectively.^37^ When the incident angle is fixed at 60° and the refractive index of the AAO layer is 1.65, the equation can be simplified to λ = 1.65d_AAO_/*m*, showing that the interference wavelength is directly related to the thickness of the AAO membranes. Because the AAO membranes are transparent, the intensity of λ_1_ is relatively low and less effective for interference. After applying a metallic coating on the surface of the AAO layer, the intensity of λ_1_ increases, enhancing the interference. Figure 1b provides side‐ and top‐view scanning electron microscopy (SEM) images of the AAO membranes, demonstrating the well‐ordered pore structure and uniform pore depth, which enhance light interference.

The applications of the patterned AAO membranes are illustrated in Figure 1c,d. Figure 1c depicts the fabrication process of the patterned AAO membranes. After the first anodization, the AAO membranes are covered by a hydrophobic dye mask, and a second anodization is then conducted. Afterward, the mask is removed with acetone to obtain the patterned AAO membranes. The pore depth of the covered and non‐covered areas differs because of the varying anodization times, leading to differences in interference wavelengths, which create patterns on the surface of the AAO membranes. Figure 1d shows the top and tilted views of photos of the patterned AAO membranes. Because of the varying light incident angle θ_i_, the interference colors change accordingly. The color appearance is also dependent on the thickness of the AAO layer, and the pore depth of AAO can be measured using SEM and other instruments. Figure 1e,f demonstrates a new method to quickly correlate anodization times with colors. By gradually lifting the foils from the electrolyte during anodization, the AAO membranes with gradient pore depths can be fabricated, allowing for the creation of a calibration curve correlating the anodization times with the colors of the AAO membranes.

Figure S1a (Supporting Information) displays the color appearance of AAO membranes fabricated using two different processes: single‐step anodization (AN1) and two‐step anodization (AN1+AN2). In the AN1+AN2 process, a chemical etching step is performed between the two anodization steps to achieve a more self‐ordered nanoporous structure on the membrane surface. Figure S1b,c (Supporting Information) presents SEM images of AAO membranes fabricated by the (b) AN1 and (c) AN1+AN2 processes, respectively. The results indicate that AAO membranes fabricated using AN1+AN2 exhibit more uniform pore sizes and arrangements. However, the color appearance of the AAO membranes remains consistent across both processes, demonstrating that pore sizes and regularities have no significant impact on the observed color. Previous studies have reported that inter‐pore distance is related to the formation and intensity of interference, which affects the development of photonic crystals.^[^ ^53^, ^54^ ^]^ Figure S1d (Supporting Information) further confirms that the inter‐pore distances of AAO membranes fabricated by both processes are similar.

**Figure 2:**
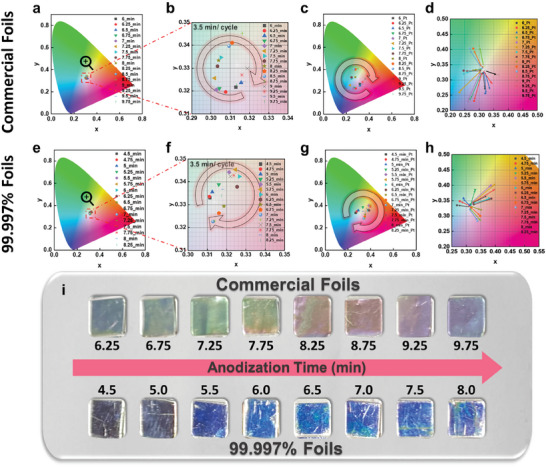
a–c) CIE 1931 color diagram of the AAO membranes with different anodization times fabricated by commercial foils: (a,b) without Pt coating and (c) with Pt coating. e–g) CIE 1931 color diagram of the AAO membranes with different anodization times fabricated from 99.997% foils: (e,f) without Pt coating and (g) with Pt coating. d,h) Enlarged CIE 1931 color diagrams of the AAO membranes fabricated from (d) commercial foils and (h) 99.997% foils without Pt coating and with Pt coating. i) Photos of the AAO membranes with different anodization times fabricated from commercial foils and 99.997% foils without Pt coating.

To investigate the effect of aluminum purity on the color appearance of AAO membranes, we use commercial aluminum foils and high‐purity (99.997%) aluminum foils. AAO membranes are fabricated by varying the anodization times, with 0.25‐min intervals between each membrane. The resulting color changes are measured using a spectrophotometer and are presented on the CIE 1931 color diagram in **Figure** **2**. Figure 2a,e shows the color appearance of the AAO membranes fabricated from commercial and 99.997% foils without Pt coating. Because of the weak intensity of interference, both types of AAO membranes exhibit a weak color appearance. The zoomed‐in CIE 1931 diagrams in Figure 2b,f further show that the color changes display counterclockwise cycles on the CIE 1931 diagram with cycle times of ≈3.5 min for both AAO membranes. The color appearance of the AAO membranes fabricated from commercial and 99.997% foils with Pt coating is shown in Figure 2c,d. With the Pt coating on the surface of the AAO membranes, the light interference increases, leading to stronger color saturation. To better compare the color appearance of the AAO membranes without Pt coating and with Pt coating, both conditions are plotted together on the CIE 1931 color diagram, as shown in Figure 2d,h. Each arrow on the diagram indicates the transition in color appearance from the AAO membranes without Pt coating to those with Pt coating. The visible light wavelength remains almost unchanged, while the color saturation becomes stronger. The photos of the AAO membranes anodized for different times, fabricated from commercial and 99.997% aluminum foils without Pt coating, are shown in Figure 2i. While all photos are captured under the same angles and conditions, the results show that the AAO membranes fabricated from commercial foils exhibit stronger color saturation compared with those from 99.997% of foils with the naked eye. However, using aluminum with excessively low purity results in poorly ordered and non‐uniform pores, which can hinder the formation of photonic crystals. The results cannot be directly compared with the spectrophotometer measurements (Figure 2b,f) because of the differences in the incident light angles. In conclusion, by using lower‐purity aluminum foils, it is possible to observe the colors formed by photonic crystals on the AAO membranes without the need for Pt coating, simplifying the fabrication process and reducing production costs.

To investigate the relationship between the anodization times and the pore depth of the AAO membranes, we fabricate AAO membranes via the two‐step anodization process using the oxalic acid electrolyte. The two‐step anodization process is better to characterize the growth rates than the one‐step anodization process because of the well‐ordered pore structure.^[^ ^23^ ^]^ The AAO membranes are anodized with varying anodization times, with 2‐min intervals between each sample. Figure S2a–h (Supporting Information) shows the side‐view SEM images of the AAO membranes at different anodization times, in which the AAO layer and the Al layer can be distinguished. The plot of pore depth versus anodization time is demonstrated in Figure S2I (Supporting Information), indicating that the pore depth increases approximately linearly with the anodization time. The growth rate of the pore depth can be calculated to be ≈1.5 µm h^−1^, which is consistent with those in previous studies.^[^ ^22^, ^55^, ^56^, ^57^ ^]^

To further investigate the differences in color appearance of the AAO membranes fabricated from commercial and 99.997% aluminum foils without Pt coating, it is crucial to understand the impurities present in both types of aluminum foils. The concentrations of impurities may affect the reflections at the AAO‐air interface, resulting in variations in interference intensity. The elemental compositions of the two types of foils are analyzed using energy‐dispersive X‐ray spectroscopy (EDS) by detecting the specific Kα values of different elements. Figure S3 (Supporting Information) shows the EDS analyses of the two types of foils, revealing Kα peaks at 0.5, 0.7, 1.5, and 1.7 keV for O, Fe, Al, and Si, respectively. It should be noted that the O Kα peak results from the natural oxidation tendency of aluminum. When calculating the aluminum content, the contribution of oxygen from the aluminum oxide (Al₂O₃) must also be taken into account. The elemental compositions of both types of foils are summarized in Table S1 (Supporting Information). The proportions of Al, Si, and Fe in the commercial foils are 97.8, 2.0, and 0.2%, respectively, while the 99.997% foils are composed almost entirely of aluminum.

**Figure 3:**
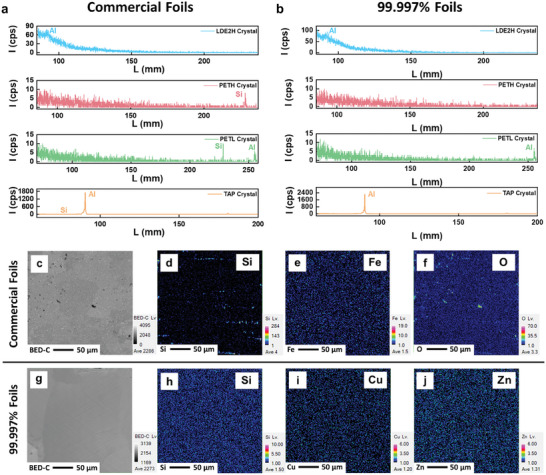
Wavelength‐dispersive X‐ray spectra (WDXS) of commercial foils a) and 99.997% foils b). Backscattered electron image c), corresponding WDXS mapping Si d), corresponding WDXS mapping Fe e), and corresponding WDXS mapping O f) of commercial foils. Backscattered electron image g), corresponding WDXS mapping Si h), corresponding WDXS mapping Cu i), and corresponding WDXS mapping Zn j) of 99.997% foils.

To obtain a deeper insight into the distribution of surface impurities and elemental compositions, field emission‐electron probe microanalysis (FE‐EPMA) combined with wavelength‐dispersive X‐ray spectroscopy (WDXS) is employed. WDXS mapping allows for the analysis of elemental distribution in smaller localized regions. By using various analyzing crystals (LDE2H, LDE1H, TAP, PETL, and PETH), it is possible to distinguish between characteristic X‐rays with similar energies through diffractions of their X‐ray lines. **Figure** **3**a,b shows the qualitative analysis of the two types of foils under four different crystals. In the LDE2H, PETL, and TAP crystal regions, both foils display Al peaks (at L = 92.6, 255.2, and 90.6 nm); however, in the PETH, PETL, and TAP crystal regions, only the commercial foils show a Si peak (at L = 228.2, 228.3, and 86.5 nm), indicating that Si is one of the major impurities.^[^ ^58^, ^59^ ^]^ Quantitative experiments are also conducted by selecting five random regions on each type of foil, and the elemental ratios are calculated and summarized in **Table** **1**. The impurities found in the commercial foils include Si, Fe, Cu, and Zn. This result is consistent with the EDS analyses, confirming that Si and Fe are the major impurities. In comparison, 99.997% of foils contain relatively low impurity levels, all under 0.1%. To further understand the impurity distribution in both types of foils, WDXS mapping analysis is conducted based on the determined elements (Si, Fe, O, Cu, and Zn) from the quantitative experiments using 250 µm × 250 µm regions, as demonstrated in Figure 3c–j. For the commercial foils, Si and O are unevenly distributed, while Fe is uniformly distributed (Figure 3d–f). For the 99.997% of foils, Si, Cu, and Fe are evenly distributed at low concentrations across the surface (Figure 3h–j). Figure S4a–e (Supporting Information) presents the XRF spectra of commercial aluminum foils and 99.997% aluminum foils. The proportions of various elements are shown in Figure S4e (Supporting Information), indicating that the primary impurities in the commercial aluminum foils are Si (1.0%) and Fe (0.4%), consistent with the FE‐EPMA results. The chemical compositions of both types of foils are summarized in Table S2 (Supporting Information). The transparency of the AAO membranes fabricated from commercial foils might be affected by the impurities. The reduced transparency of the AAO membranes could enhance the reflectance at the AAO‐air interface, increasing the intensity of interference and resulting in a higher color appearance for the AAO membranes fabricated from commercial foils without Pt coating. In contrast, for the AAO membranes fabricated from the 99.997% foils without Pt coating, the lower impurity content, even though evenly distributed, does not significantly reduce the transparency of the AAO membranes, resulting in a weaker color appearance.

**Table 1:** Chemical compositions of commercial and 99.997% foils by WDXS measurements.

	Al	Si	O	Fe	Zn	Cu
Commercial Foil	94.7	2.6	2.4	0.3	<0.1	<0.1
99.997% Foil	99.9	–	–	–	<0.1	<0.1

The effect of inter‐pore distance on the color appearance of AAO membranes is also investigated. Figure S5a,b (Supporting Information) illustrates the color appearance of AAO membranes without and with Pt coating using sulfuric acid as the electrolyte. The results show that the visible color disappears after 15 min of anodization. It is worth noting that, under the same anodization period, the color changes of the AAO membranes using sulfuric acid as the electrolyte occur faster than those using oxalic acid. This result may be attributed to the different pore depth growth rates associated with these electrolytes.^[^ ^60^ ^]^ Figure S5c,d (Supporting Information) presents SEM images of the AAO membranes fabricated using (c) sulfuric acid and (d) oxalic acid as electrolytes. Image analysis using ImageJ reveals that the inter‐pore distances of AAO membranes fabricated with sulfuric acid and oxalic acid electrolytes are 47.4 and 30.3 nm, respectively, as summarized in Figure S5e (Supporting Information). To further investigate the color appearance of AAO membranes using sulfuric acid as the electrolyte, both with and without Pt coating, the CIE 1931 color diagram, measured using a spectrophotometer, is shown in Figure S5f,g (Supporting Information). The data indicate that the color remains the same after 12 min of anodization, consistent with visual observations. Additionally, Figure S5h (Supporting Information) demonstrates that the color saturation of AAO membranes fabricated with oxalic acid is higher than that with sulfuric acid. This observation suggests that oxalic acid is a more suitable electrolyte for producing colorful AAO membranes.

**Figure 4:**
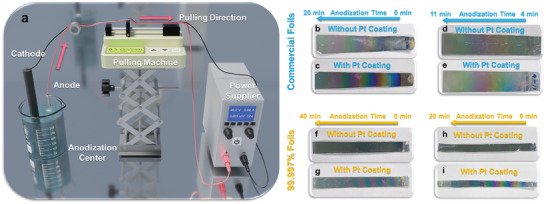
a) Schematic illustration of the experimental setup of the AAO membranes with gradient pore depths. b,c) Photos of the AAO membranes with 0 to 20 min anodization times fabricated by commercial foils without (b) and with (c) Pt coating. d,e) Photos of the AAO membranes with 4 to 11 min anodization times fabricated by commercial foils without (d) and with (e) Pt coating. f,g) Photos of the AAO membranes with 0 to 40 min anodization times fabricated by 99.997% foils without (f) and with (g) Pt coating. h,i) Photos of the AAO membranes with 0 to 20 min anodization times fabricated by 99.997% foils without (h) and with (i) Pt coating.

After confirming the impurities of the aluminum foils, we develop a promising approach to control the colors of the AAO membranes and measure the correlation between the colors and the anodization times. The designed experimental setup is illustrated in **Figure** **4**a. A pulling machine is positioned between the electropolished aluminum foils and the power supply with the anode electric wires attached to the pulling machine, allowing the foils to be gradually lifted from the electrolytes during the anodization process. Different durations of the gradient anodization times can be achieved by adjusting the pulling speed of the machine. This method enables the fabrication of AAO membranes with gradient pore depths, producing colorful membranes. Furthermore, this approach allows for the correlation between the anodization times and the colors of the AAO membranes, enabling the creation of a calibration curve for the production of specific colors on AAO membranes. As a result, AAO membranes with different durations of gradient anodization times with and without Pt coating are fabricated, as shown in Figure 4b‐i. Figure 4b,c and Figure 4d,e show the AAO membranes with gradient pore depths fabricated from the commercial foils with anodization times ranging from 0 to 20 min and 4 to 11 min, both without and with Pt coating, respectively. The color changes can be observed as the anodization time increases, and by shortening the anodization time range, finer color variations can be achieved. Figure 4f,g shows an AAO membrane with a longer anodization time range, where the color appearance begins to fade after 30 min of anodization. Additionally, the color appearance exhibits cycles between 10 and 30 min of anodization. It is worth noting that, under the condition without Pt coating, the AAO membranes produced from the commercial foils display better surface coloration (Figure 4b,d). In contrast, the AAO membranes made from 99.997% foils show minimal surface colors (Figure 4f,h). Moreover, commercial foils offer the advantages of low cost and high availability, making them suitable for large‐scale applications. For this reason, the commercial foils rather than the 99.997% foils are selected in the subsequent experiments, in which step of Pt coating is not required.

**Figure 5:**
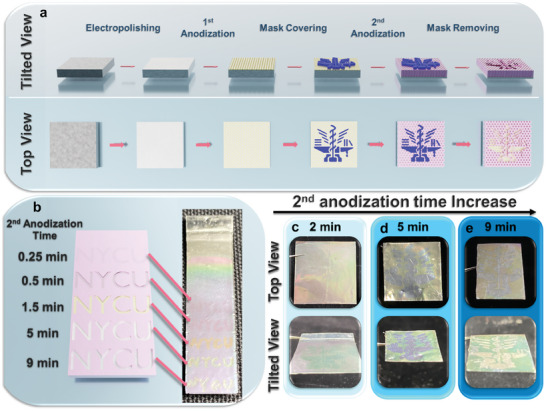
a) Schematic illustration of the fabricating process of the patterned AAO membranes. b) Graphical illustration and photos of the AAO membranes with patterns fabricated with second anodization times from 0.25 to 9 min. c–e) Top‐ and tilted‐view photos of the patterned AAO membranes with patterns fabricated with anodization intervals of (c) 2, (d) 5, and (e) 9 min.

To demonstrate the practical applications of the colored AAO membranes, we further fabricate patterned AAO membranes for anticounterfeiting using the two‐step anodization procedure, as illustrated in **Figure** **5**a. The commercial aluminum foils are first cleaned with acetone, ethanol, and isopropanol to remove organic impurities, followed by electropolishing to eliminate surface contaminants. Afterward, the first anodization is performed using oxalic acid as the electrolyte to produce AAO membranes with the first color (Color A). A hydrophobic mask is then applied to the surface, and the second anodization is carried out to generate AAO membranes with the second color (Color B). After the mask is removed, patterned AAO membranes containing both colors A and B can be obtained. During the fabrication process, the durations of the first and second anodization are adjusted individually, while maintaining the total anodization (first and second anodization) times at 10 min. Five different first/second anodization times (1/9, 4/5, 8.5/1.5, 9.5/0.5, and 9.75/0.25 min min^−1^) are applied to create the patterned AAO membranes on the same piece of commercial foils, as shown in Figure 5b. The results indicate that shorter second anodization times lead to more similar colors between the masked region (Color A) and unmasked region (Color B), resulting in less distinct patterns. AAO membranes with more complex patterns are shown in Figure 5c–e. The results demonstrate that viewing the patterned AAO membranes from different angles changes the clarity of the patterns. This phenomenon occurs when viewed from the top (top‐view), and the interference intensity is weaker due to the alignment of light paths, making the patterns less visible. Conversely, when viewed from an angle (tilted view), the interference becomes stronger, enhancing the visibility of the patterns. This angle‐dependent optical effect adds a layer of complexity, making replication more difficult and thus enhancing the anti‐counterfeiting capability. It should be noticed that the patterns produced with a 2‐min second anodization time (Figure 5c) are almost invisible from the top view but visible when observed at tilted angles. The results suggest that patterned AAO membranes could have potential for use in anticounterfeiting applications.

**Figure 6:**
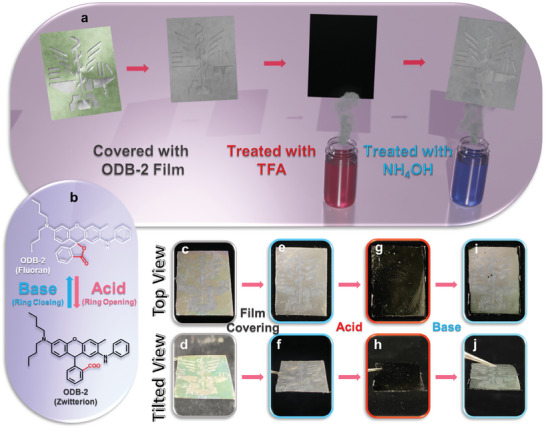
a) Schematic illustration of the pH‐responsive anticounterfeiting AAO membranes. b) Ring‐opening and the ring‐closing reaction of ODB‐2 through pH changes. c,e,g,i) Top‐view photos of the responsive anticounterfeiting AAO membranes: c) patterned AAO membranes, e) patterned AAO membranes covered with ODB‐2 films, g) patterned AAO membranes after acid (TFA) treatment, and i) patterned AAO membranes after base (NH₄OH) treatment. d,f,h,j) Tilted‐view photos of the responsive anticounterfeiting AAO membranes: (d) patterned AAO membranes, (f) patterned AAO membranes covered with ODB‐2 films, (h) patterned AAO membranes after acid treatment, and (j) patterned AAO membranes after base treatment.

To further explore the functionality of the patterned AAO membranes, we also develop responsive anticounterfeiting AAO membranes by integrating responsive materials, as illustrated in **Figure** **6**a. A 2‐anilino‐6‐dibutylamino‐3‐methylfluoran (ODB)‐2/ thermoplastic polyurethane (TPU) solution (4 wt% ODB‐2 and 4 wt% TPU in THF) is coated on the patterned AAO membranes using a spin‐coating process. ODB‐2 is a responsive color‐changing material, with the color change influenced by pH values, while TPU is a flexible polymer that provides both flexibility and stretchability to the film. After being covered with the ODB‐2/TPU film, the color appearance of the patterned AAO membranes changes, due to different interference caused by the additional ODB‐2/TPU layer; however, the patterns can still be distinguished. By further exposing the covered AAO membranes to TFA vapors, an acidic environment is created, causing the membranes to turn black and disappearance of the patterns; on the contrary, by exposing the covered AAO membranes to NH_4_OH vapors, an alkaline environment is created, causing the membranes turning transparent and reappearance of the patterns.

The color‐changing mechanism of ODB‐2 is displayed in Figure 6b, where the ODB‐2 molecule undergoes a ring‐opening reaction upon pH changes, transitioning from the lactone form (colorless) to the zwitterionic form (colored). Figure 6c–j shows photos of the responsive patterned AAO membranes from both top‐ and tilted views, demonstrating the pattern changes after different treatments. Specifically, after being treated with TFA vapors, the ODB‐2/TPU film turns black and the patterns become unrecognizable (Figure 6g,h). The times required for the color changes are ≈1 and ≈10 s for acidic and alkaline treatments, respectively. Table S3 (Supporting Information) also shows the response times of the ODB‐2/TPU films under different acidic and basic conditions. From the results, the response times for hydrochloric acid and trifluoroacetic acid are less than 1 s, while the acetic acid is approximately 15 s; the response times for triethylamine, pyridine, and ammonium hydroxide are 91, 39, and 10 s, respectively.

To further study the reliability of the responsive patterned AAO membranes, repeated acid/base vapor treatments are conducted, as presented in Figure S6b (Supporting Information). The absorbance at 610 nm remains consistent across 10 cycles, demonstrating the reversibility and stability of the pH‐responsive behavior. These findings highlight that the ODB‐2/TPU films are a promising candidate for anticounterfeiting applications with multiple uses. Overall, the results indicate that the responsive patterned AAO membranes are suitable for anticounterfeiting applications in varying pH environments. The ODB‐2/TPU‐coated AAO membranes exhibit a pH‐responsive mechanism with response times notably faster than other reported materials, as summarized in Table S4 (Supporting Information).

Furthermore, to better illustrate the practical utility of our pH‐responsive AAO membranes, we have tested their application on various real‐world items, as shown in Figure S7 (Supporting Information). The membranes are applied to a computer motherboard (Figure S7a,b, Supporting Information), the corner of a certificate (Figure S7c,d, Supporting Information), and a student ID card (Figure S7e,f, Supporting Information). These examples showcase the versatility of our materials in various anticounterfeiting scenarios, particularly when integrated into real‐world items such as certificates and ID cards, highlighting their potential to enhance security while remaining cost‐efficient. Moreover, because of their rapid responses to acidic and alkaline conditions, the AAO membranes are suitable for integration into safety stickers or labels. Under acidic environments, these membranes can immediately display visible color changes, providing a fast and intuitive method for detecting hazardous conditions.

**Figure 7:**
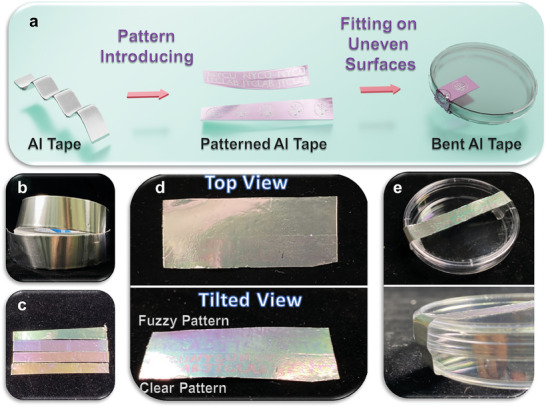
a) Schematic illustration of the patterned AAO membranes fabricated by aluminum tapes. b) Photos of aluminum tapes. c) Photos of the AAO membrane tapes with different colors. d) Top‐ and tilted‐view photos of the patterned AAO membrane tapes. e) Photo of a bent patterned AAO membrane tape attached to a Petri Dish.

To further extend the applications of the patterned AAO membranes, we also fabricate flexible anticounterfeiting stickers using aluminum tapes as the substrates for AAO membranes, as illustrated in **Figure** **7**a. Compared with aluminum foils (thickness: ≈100 µm), aluminum tapes (thickness: 22 40 µm) are much more flexible, enabling the bending of the patterned AAO membranes. The flexibility also allows better attaching of the membranes on non‐flat surfaces or wearable devices. As a result, bendable and patterned AAO membrane tapes are highly versatile and capable of fitting onto uneven surfaces. Figure 7b shows a photo of the original commercial aluminum tape used in this work. By controlling the anodization times to 8, 9, 10, and 11 min, AAO membrane tapes with different colors can be fabricated, as shown from bottom to top in Figure 7c. The results indicate that the color appearances of the AAO membranes fabricated on the aluminum tapes are comparable to those of those on the commercial foils. Patterned AAO membranes are further prepared using aluminum tapes, as shown in the top‐ and tilted‐view photos (Figure 7d). The second anodization times for the upper and lower parts of the patterned AAO membrane tape are 2 and 5 min, respectively. The result demonstrates that clearer patterns can be observed from the tilted view than those from the top view because of the different interference intensities. Besides, the patterns with longer second anodization times show higher clarity, caused by the larger pore depth differences. The flexibility of the patterned AAO membrane tapes is also demonstrated by attaching them to Petri Dish (Figure 7e), highlighting their potential as security packaging tapes and various anti‐counterfeiting applications. It is also important to note that, by appropriately adjusting the duration of the second anodization step, the generated patterns are less noticeable from the top view but become much clearer when observed from a tilted view. This optical phenomenon arises from the interference effects caused by varying pore depths, achieved through the principles of photonic crystals. Unlike the pH‐responsive anticounterfeiting mechanism demonstrated in Figure 6, the anticounterfeiting effect of the Al tapes only relies on structural interference and does not involve any responsiveness.

The mechanical properties of the patterned AAO membrane tapes are evaluated to demonstrate their stability and durability, as shown in Figure S8 (Supporting Information). The mechanical results indicate consistent performance across three tests with an average maximum stress of 65.0 MPa and an average elongation of 7.5%. These findings demonstrate that the patterned Al tapes exhibit good mechanical properties, making them suitable for practical applications. Furthermore, due to the stability of the anodized aluminum layer, the patterned surface is resistant to excessive oxidation over long‐term storage, ensuring that the patterns remain intact. This robustness and durability highlight the potential of the patterned Al tape as a reliable material for anticounterfeiting packaging labels. The mechanical data are summarized in Table S5 (Supporting Information).

## 3 Conclusion

In conclusion, we demonstrate the potential of anodic aluminum oxide (AAO) membranes for anticounterfeiting applications by simplifying the fabrication process while maintaining effective color generation without metal coatings. Unlike traditional methods requiring metal layers, we use commercial aluminum foils to produce colorful AAO membranes, reducing both the cost and complexity of the production process. By integrating a novel method for fabricating AAO membranes with gradient pore depths, we establish a correlation between anodization time and color, providing further control over the material's properties. The two‐step anodization process, combined with a hydrophobic mask, enables precise patterning on the membranes, enhancing their anticounterfeiting potential. Moreover, the integration of pH‐responsive ODB‐2/TPU films further expands the versatility of these membranes by allowing for responsive color changes under varying pH conditions. While traditional methods often rely on surface modifications or complex chemical treatments, this work simplifies the fabrication process by employing a two‐step anodization technique to directly create customizable patterns on the AAO membranes. This approach reduces both the complexity and cost of production while maintaining high levels of security and functionality, offering significant advancements in AAO membrane applications for anticounterfeiting.

## 4 Experimental Section

### Materials

Sulfuric acid (95.0–97.0%), perchloric acid (70.0–72.0%), and ethanol (>99.8%) were obtained from Honeywell. Oxalic acid (anhydrous, 97.0%) was bought from Showa. Potassium dichromate (99.0%) was purchased from Alfa Aesar. Isopropanol (IPA, 99.5%) was acquired from Echo Chemical. Acetone (HPLC/spectroscopy grade, 99.9%) and tetrahydrofuran (THF) were obtained from Tedia. Trifluoroacetic acid (TFA) and ammonium hydroxide (28.0–30.0%) were supplied by Thermo Scientific and J. T. Baker, respectively. Thermoplastic polyurethane (TPU, grade 1685A‐E2) was bought from the Great Eastern Resin Industry. 2‐Anilino‐6‐dibutylamino‐3‐methylfluoran (ODB‐2) was acquired from Combi‐Blocks. High‐purity aluminum sheets (thickness: 0.2 mm; 99.997%) were acquired from Alfa Aesar. Commercial aluminum foils (thickness: 0.1 mm; 98%) were purchased from Kunshan Aluminum. Microscope glass slides were obtained from DGS.

### Fabrication of the AAO Membranes with and Without Pt Coating

Initially, the aluminum foils were cut into 1 × 4 cm^2^ pieces. The foils were then sequentially cleaned with soapy water, acetone, ethanol, and isopropanol in an ultrasonic bath for 3 min to remove organic impurities, grease, and surface particles. After cleaning, the aluminum foils were electropolished in a solution consisting of 80 vol % ethanol and 20 vol % perchloric acid at 20 V and 0 °C for 5 min to eliminate surface contaminants. Subsequently, the electropolished aluminum foils were immersed in 0.3 m oxalic acid or 0.3 m sulfuric acid at 0 °C, undergoing anodization at 40 and 25 V, respectively, for 0.5 to 10 min to form AAO membranes. During anodization, the current was carefully controlled and maintained below a maximum of 0.1 A to ensure operational safety. The process was performed in a controlled environment at 25 °C to maintain consistency and reproducibility. After anodization, the membranes were dried in vacuum desiccators. Thin platinum (Pt) layers (≈4 nm) were then coated onto the AAO membranes using a sputter coater (JEOL JFC‐1600) with a 20 mA current for 50 s to enhance surface reflectivity. Finally, the AAO membranes with varying anodization times were obtained. The fabrication of the AAO membranes without Pt coating followed the same procedure, excluding the Pt coating step.

### Fabrication of the AAO Membranes with Gradient Pore Depths with and Without Pt Coating

In the beginning, the aluminum foils were cut into 1 × 7 cm^2^ pieces. The cleaning and electropolishing methods for the foils followed the same procedures as those described for the fabrication of AAO membranes with and without Pt coating. After electropolishing, a pulling machine was positioned between the aluminum foils and the power supply with the anode electric wires attached to the pulling machine. The foils were then immersed in 0.3 m oxalic acid at 0 °C, undergoing anodization at 40 V while the pulling machine continuously lifted the foils from the electrolyte. The pulling speeds were set at 0.15, 0.3, and 0.9 cm/min to produce AAO membranes for different gradient anodization times. After anodization, the membranes were dried in vacuum desiccators. A thin platinum layer (≈4 nm) was subsequently coated on the AAO membranes using a sputter coater with a 20 mA current for 50 s to enhance surface reflectivity. The fabrication of AAO membranes with gradient pore depths without Pt coating followed the same procedure, excluding the Pt coating step.

### Fabrication of the Anticounterfeiting AAO Membranes

First, the aluminum foils were cut into 3 × 3 cm^2^ pieces. The foils were cleaned and electropolished using the same methods as for the AAO membranes with and without Pt coating. Subsequently, the foils underwent the first anodization using 0.3 m oxalic acid as an electrolyte at 0 °C and 40 V for 1 to 9.75 min to obtain the first‐anodized AAO membranes. Then, the hydrophobic dye was applied on the surface of the first‐anodized AAO membranes to form the patterns. Later, the patterned foils underwent a second anodization using the same condition as the first anodization for 9 to 0.25 min. It should be noted that the total anodization times were controlled at 10 min. Finally, the AAO membranes were washed with acetone to remove the hydrophobic dye, obtaining the patterned AAO membranes.

### Fabrication of the Responsive ODB‐2 Films

First, thermoplastic polyurethane (TPU, *M*_w_: 110 kg mol^−1^) and 2‐anilino‐6‐dibutylamino‐3‐methylfluoran (ODB‐2) were dissolved in tetrahydrofuran (THF) at various concentrations. The weight percentages of TPU and ODB‐2 ranged from 0–10 wt% and 0.5–10 wt%, respectively. These mixtures were stirred at 150 rpm and 40 °C for 10 h to obtain the homogeneous solutions. Subsequently, the mixture solutions were coated onto the patterned AAO membranes using a spin coater with a spinning rate of 5000 rpm for 30 s to obtain the responsive ODB‐2 films. Finally, the polymer films on the patterned AAO membranes were dried in vacuum desiccators to eliminate residual THF and stored in ambient conditions for further use.

### Analysis and Characterization

The surface morphologies of the AAO membranes were examined using a scanning electron microscope (SEM, JEOL JSM‐7401) at an acceleration voltage of 5 kV. Before SEM analyses, the AAO membranes were dried in vacuum desiccators and coated with a thin Pt layer (≈4 nm) using a sputter coater (JEOL JFC‐1600) at a 20 mA current for 50 s to enhance conductivity. The chemical properties of the aluminum foils and AAO membranes were then assessed through multiple analytical techniques, including wavelength‐dispersive X‐ray spectroscopy (FE‐EPMA, JEOL JXA‐8530F Plus), energy dispersive spectroscopy (EDS, Oxford EDS 7585), and X‐ray fluorescence spectroscopy (XRF, Hitachi EA1400). Reflectance spectroscopy measurements of the AAO membranes were performed using a Konica Minolta CM‐5 spectrophotometer, covering a wavelength range of 360–740 nm. For quantitative analysis of the AAO membranes, ImageJ software was employed.

## Conflict of Interest

The authors declare no conflict of interest.

## Supporting information



Supporting Information

## Data Availability

The data that support the findings of this study are available from the corresponding author upon reasonable request.
