# Supplementary material for: A Streamlined Approach to Anticounterfeiting Technologies: Patterned AAO Membranes Based on Photonic Crystal Effects with Tunable Color Shifts and pH Responsiveness
Source: Small. 2025 Jan 14;21(8):2409919. doi: 10.1002/smll.202409919 (PMC11855261; doi:10.1002/smll.202409919)
Supplement: Supplementary file 1 — Supporting Information [file SMLL-21-2409919-s001.docx]

**Supporting Information**

**A Streamlined Approach to Anti-Counterfeiting Technologies: Patterned AAO Membranes Based on Photonic Crystal Effects with Tunable Color Shifts and pH Responsiveness**

Yu-Chun Lin,^1^ Lin-Ruei Lee,^1^ Tsung-Hung Tsai,^1^ Ji Lin,^1^ Yen-Shen Hsu, ^1^ Manibalan Kesavan,^1^ Yu-Liang Lin,^1^ Yi-Fan Chen,^1^ and Jiun-Tai Chen^*12^

^1^Department of Applied Chemistry, National Yang Ming Chiao Tung University, Hsinchu, Taiwan 300093.
^2^Center for Emergent Functional Matter Science, National Yang Ming Chiao Tung University, Hsinchu, Taiwan 300093.

*To whom correspondence should be addressed. E-mail: jtchen@nycu.edu.tw. Tel.: +886-3-5731631


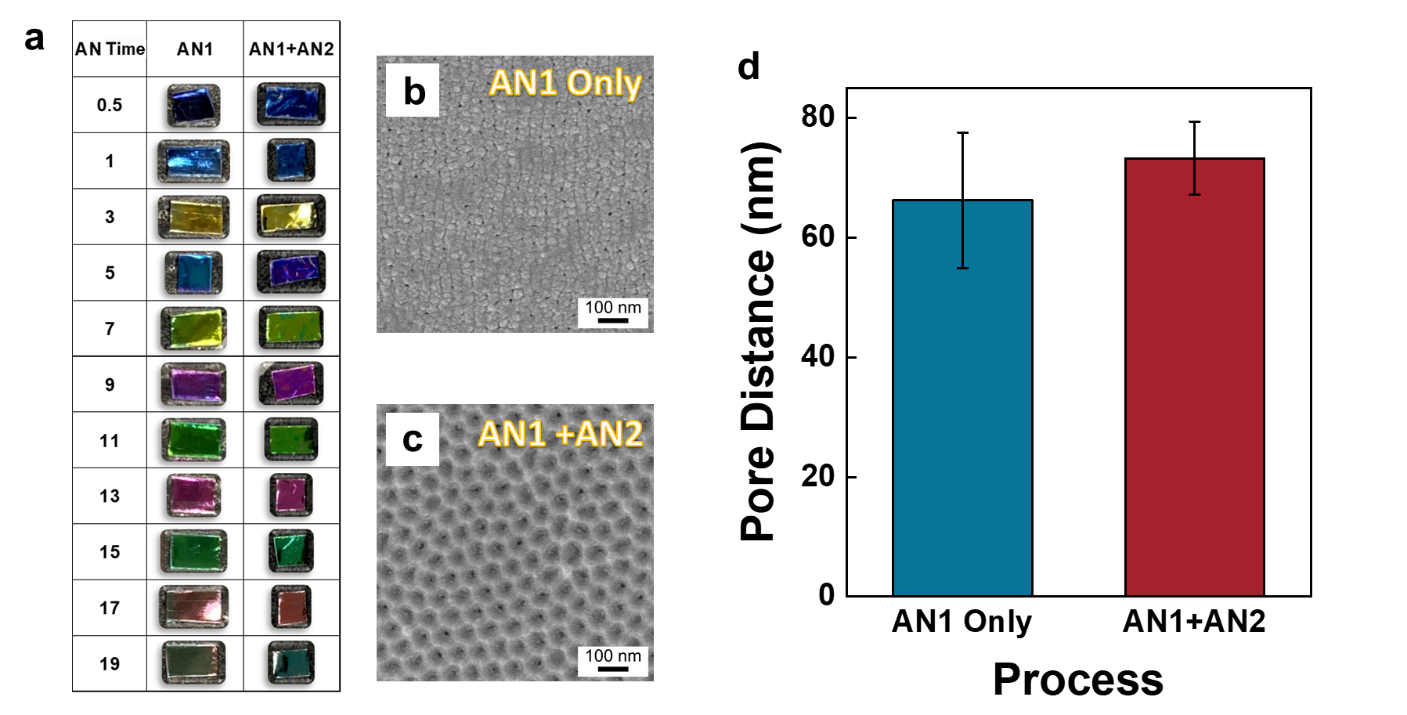


**Figure S1.** (a) Photos of AAO membranes with different anodization times fabricated by single step anodization (AN1) and 2 step anodization (AN1+AN2) processes. (b,c) SEM images of AAO membranes fabricated by (b) AN1 and (c) AN1+AN2 processes. (d) Plot of pore distances of the AAO membranes fabricated by AN1 and AN1+AN2 processes.


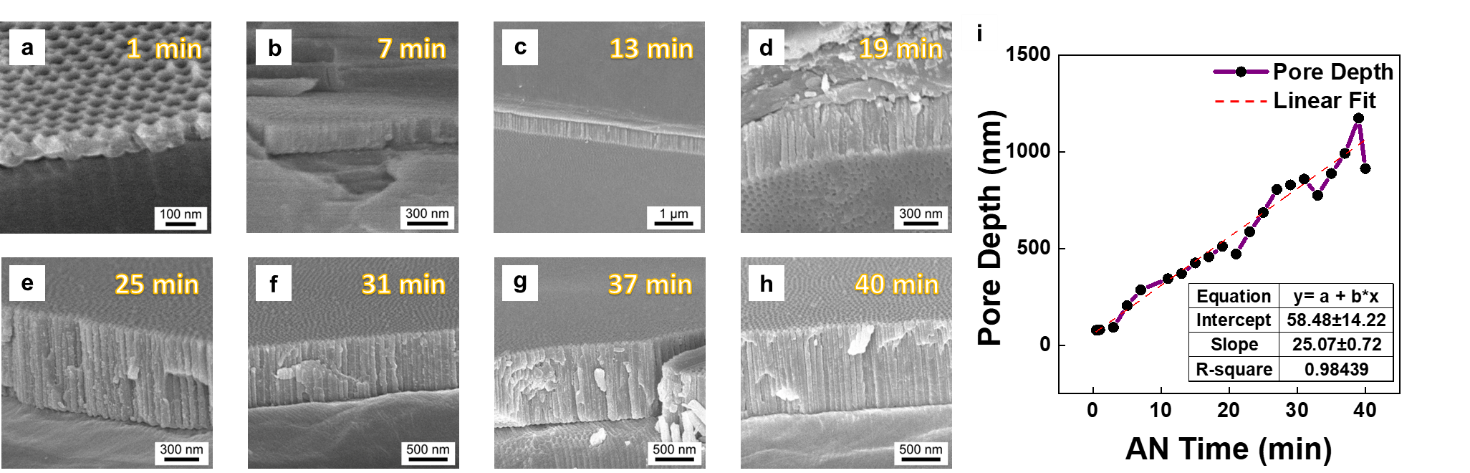


**Figure S2.** (a-h) SEM images of the AAO membranes for different anodization times: (a) 1, (b) 7, (c) 13, (d) 19, (e) 25, (f) 31, (g) 37, and (h) 40 min. (i) Plots of pore depth of the AAO membranes at different anodization times.


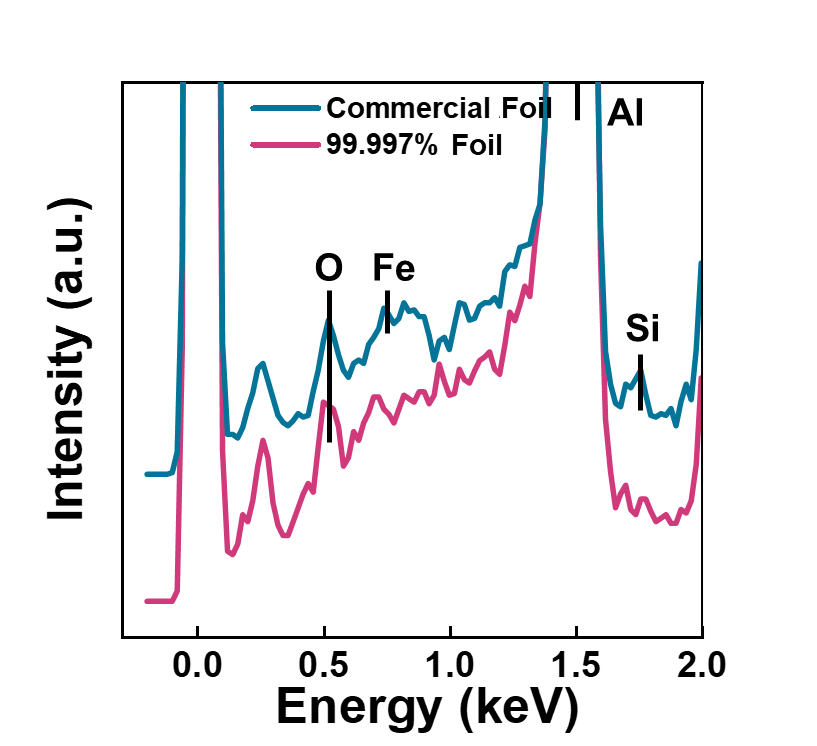


**Figure S3.** EDX spectra of commercial and 99.997% foils.

**Table S1.** Chemical compositions of commercial and 99.997% foils by EDX measurements

**Unit: wt %**

| **Element** | **Al** | **O** | **Si** | **Fe** |
| --- | --- | --- | --- | --- |
| **Commercial Foil** | 84.9 | 12.9 | 2.0 | 0.2 |
| **99.997% Foil** | 89.1 | 10.9 | - | - |


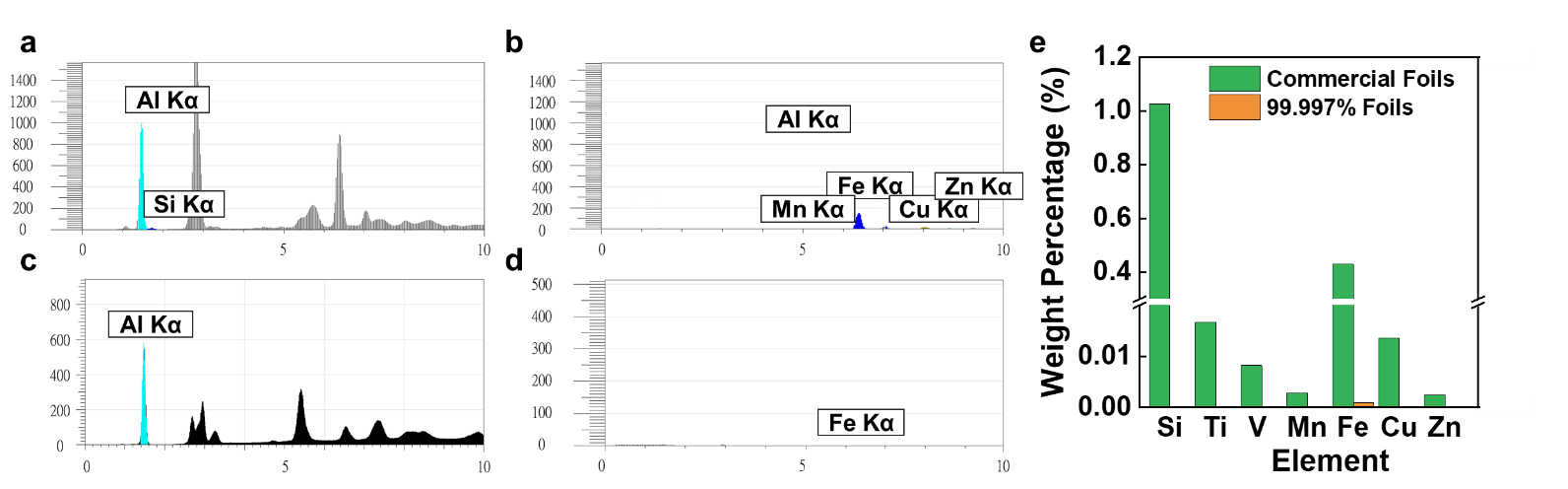


**Figure S4.** XRF spectra of (a,b) commercial aluminum foils and (c,d) 99.997% aluminum foils. (e) Statistical analyses of the weight percentages of elements in commercial and 99.997% aluminum foils.

**Table S2.** Chemical compositions of commercial and 99.997% aluminum foils by XRF measurements

**Unit: wt %**

| **Elements** | **Al** | **Si** | **Ti** | **V** | **Mn** | **Fe** | **Cu** | **Zn** |
| --- | --- | --- | --- | --- | --- | --- | --- | --- |
| **Commercial Foil** | 98.5 | 1.0 | <0.1 | <0.1 | <0.1 | 0.4 | <0.1 | <0.1 |
| **99.997% Foil** | 99.9 | - | - | - | - | <0.1 | - | - |


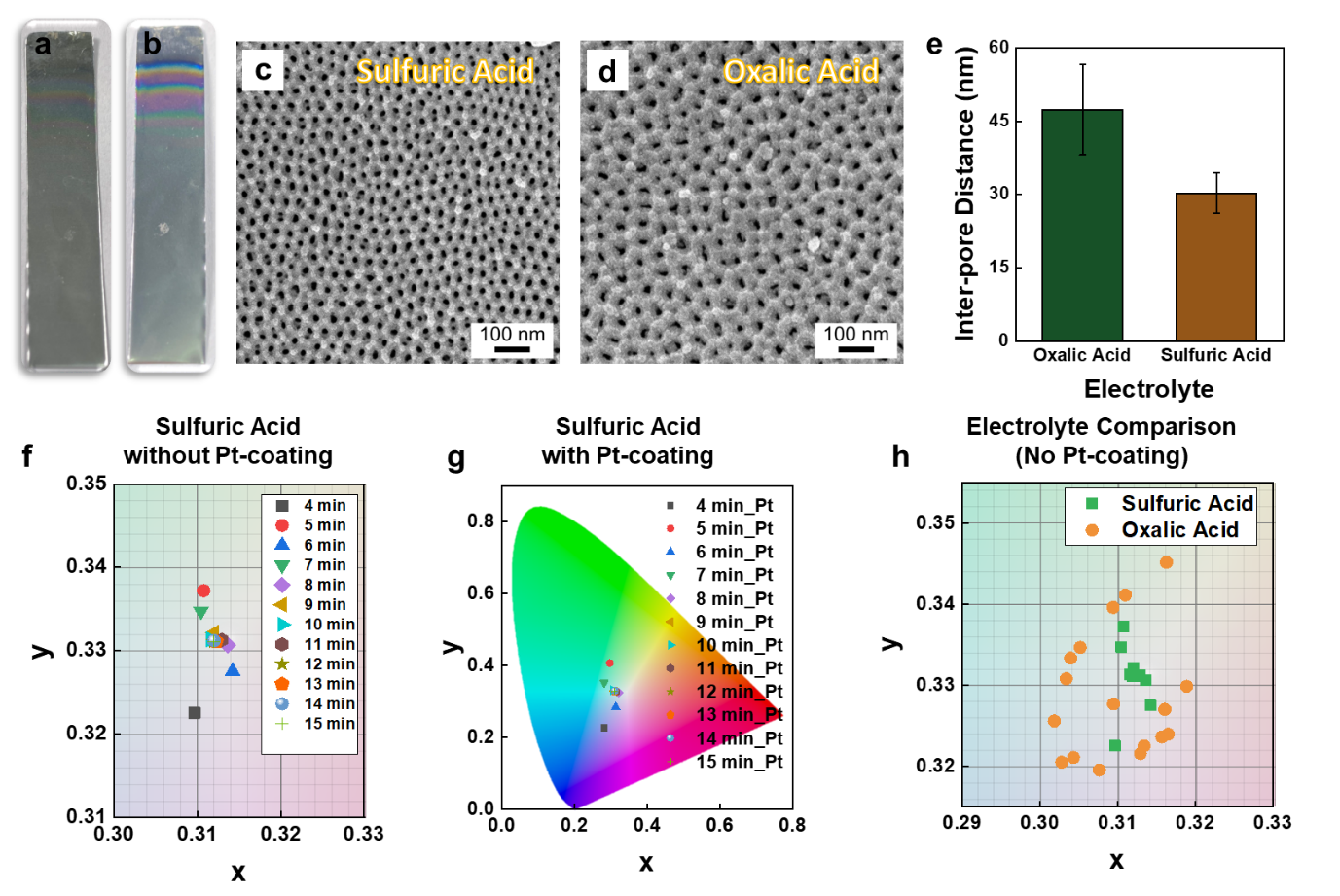


**Figure S5.** (a,b) Photos of the AAO membranes using sulfuric acid as the electrolyte with 0 to 40 min anodization time (a) without and (b) with Pt coating. (c,d) SEM images of the AAO membranes using (C) sulfuric acid and (d) oxalic acid as the electrolytes. (e) Plot of inter-pore distance of the AAO membranes using oxalic acid and sulfuric acid as the electrolytes. (f,g) CIE 1931 color diagram of the AAO membranes using sulfuric acid as the electrolyte with different anodization times (f) without and (g) with Pt coating. (h) Color comparison of the AAO membranes using oxalic acid and sulfuric acid as the electrolytes on the CIE 1931 color diagram.


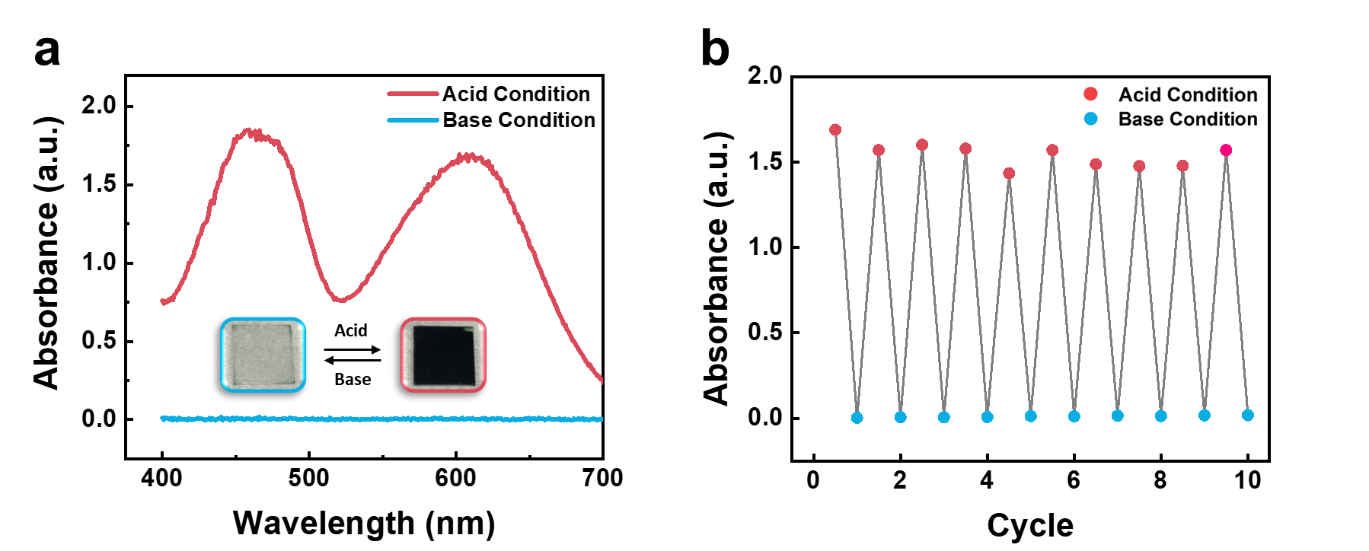


**Figure S6. (**a) UV-vis spectra of the ODB-2/TPU film under acidic/basic vapors treatment. (b) Plot of the absorbance at 610 nm versus the numbers of the cycles of acidic/basic vapors treatment of the ODB-2/TPU films.

**Table S3.** The response time of the ODB-2/TPU films under different acidic and basic conditions

| **Materials** | **Hydrochloric Acid** | **Trifluoroacetic Acid** | **Acetic Acid** | **Triethylamine** | **Pyridine** | **Ammonium Hydroxide** |
| --- | --- | --- | --- | --- | --- | --- |
| **Time (s)** | <1 | <1 | 15 | 91 | 39 | 10 |


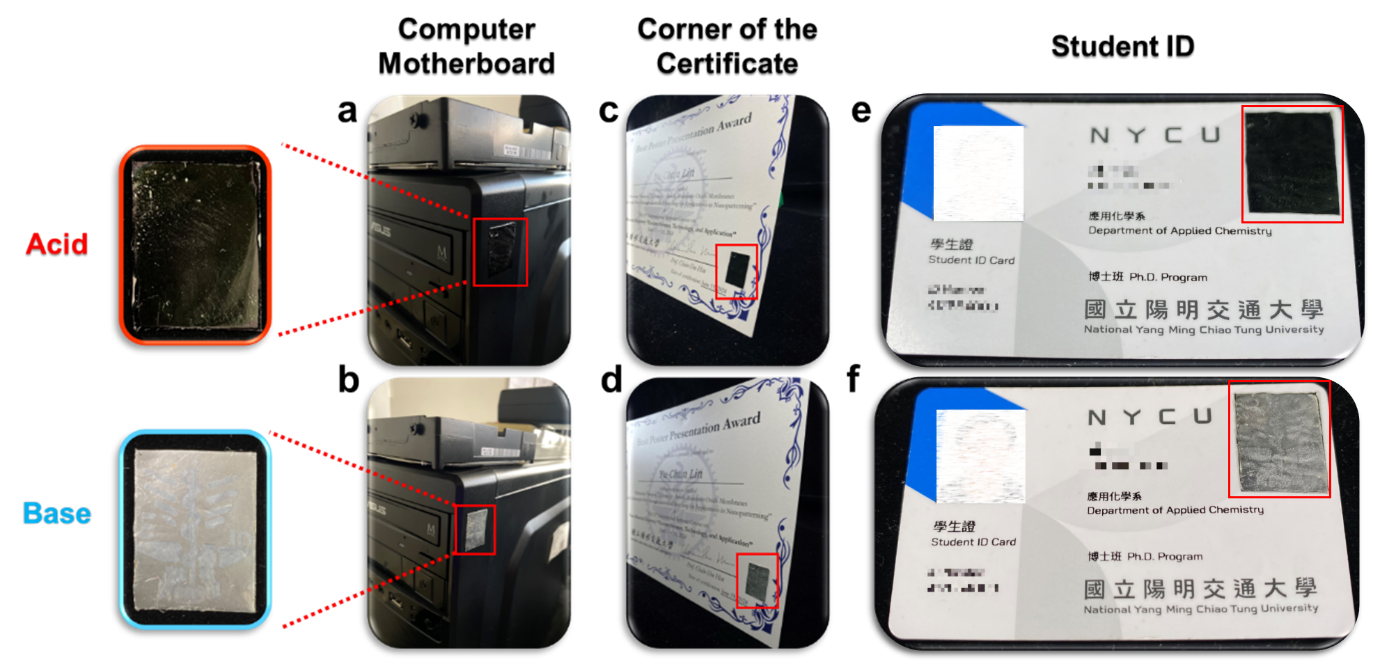


**Figure S7.** Photos of the pH-responsive anticounterfeiting AAO membranes attached on (a,b) computer motherboard, (c,d) certificate, and (e,f) student ID.

**Table S4.** Comparison of responsive anti-counterfeiting systems, including this work and representative studies from the literature

| **Article** | **Material** | **Responsive type** | **Responsive time** | **Description** |
| --- | --- | --- | --- | --- |
| This work | AAO/ODB2 | pH | ~10 s | Patterned AAO membranes coated with an ODB/TPU film exhibiting pH-chromic behavior, where the display of the pattern can be controlled by the pH of the environment. |
| 1 | PMMA/Spiropyran | Light | 100-200 s | Rewritable anticounterfeiting inks based on functionalized stimuli-responsive latex particles containing spiropyran |
| 2 | UCNPs/Dye | Light/pH | 10-100 s | Dual stimuli-responsive inks combining pH-sensitive dyes and orthogonal upconversion nanomaterials |
| 3 | N,S-CDs/Eu-MOF | pH | ~300 s | Dual-emission composite N,S-CDs@Eu-MOF with reversible fluorescence switching under acid and base vapors |


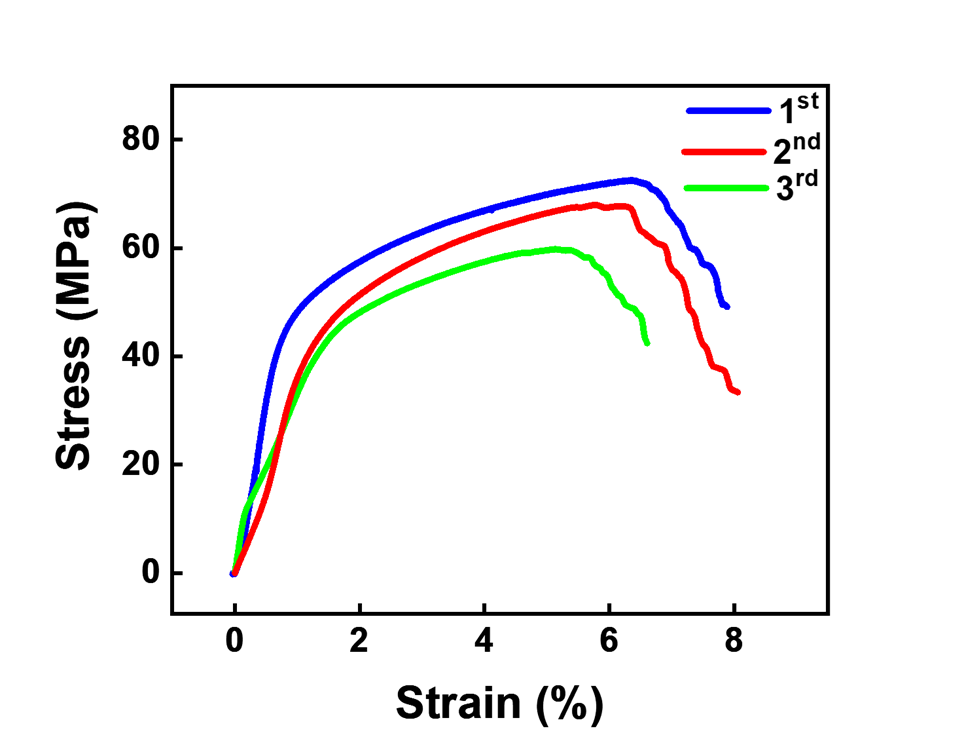


**Figure S8.** Stress versus strain (S-S) curves of Al tapes from 3 independent stretching tests.

**Table S5.** Max MPa and Max strain of patterned Al tapes in 3 independent tests

|  | **1^st^** | **2^nd^** | **3^rd^** | **Mean** |
| --- | --- | --- | --- | --- |
| **Max MPa (MPa)** | 67.1 | 67.9 | 59.8 | 65.0 |
| **Max strain (%)** | 7.9 | 8.1 | 6.6 | 7.5 |

**REFERENCES**

(1) Abdollahi, A.; Sahandi-Zangabad, K.; Roghani-Mamaqani, H. Rewritable Anticounterfeiting Polymer Inks Based on Functionalized Stimuli-Responsive Latex Particles Containing Spiropyran Photoswitches: Reversible Photopatterning and Security Marking. *ACS Appl. Mater. Interfaces* **2018**, *10*, 39279-39292.

(2) Jia, H.; Teng, Y.; Li, N.; Li, D.; Dong, Y.; Zhang, D.; Liu, Z.; Zhao, D.; Guo, X.; Di, W.; et al. Dual Stimuli-Responsive Inks Based on Orthogonal Upconversion Three-Primary-Color Luminescence for Advanced Anticounterfeiting Applications. *ACS mater. lett.* **2022**, *4*, 1306-1313.

(3) Gao, J.-P.; Yao, R.-X.; Chen, X.-H.; Li, H.-H.; Zhang, C.; Zhang, F.-Q.; Zhang, X.-M. Blue luminescent N,S-doped carbon dots encapsulated in red emissive Eu-MOF to form dually emissive composite for reversible anti-counterfeit ink. *Dalton Trans.* **2021**, *50*, 1690-1696.
